# Chloroplast Genome Evolution in Actinidiaceae: *clpP* Loss, Heterogenous Divergence and Phylogenomic Practice

**DOI:** 10.1371/journal.pone.0162324

**Published:** 2016-09-02

**Authors:** Wen-Cai Wang, Si-Yun Chen, Xian-Zhi Zhang

**Affiliations:** 1 College of Forestry, Northwest A&F University, Yangling, Shaanxi, China; 2 School of Biological and Chemical Sciences, Queen Mary University of London, Mile End Road, London, United Kingdom; 3 Germplasm Bank of Wild Species, Kunming Institute of Botany, Chinese Academy of Sciences, Kunming, Yunnan, China; University of Western Sydney, AUSTRALIA

## Abstract

Actinidiaceae is a well-known economically important plant family in asterids. To elucidate the chloroplast (cp) genome evolution within this family, here we present complete genomes of three species from two sister genera (*Clematoclethra* and *Actinidia*) in the Actinidiaceae via genome skimming technique. Comparative analyses revealed that the genome structure and content were rather conservative in three cp genomes in spite of different inheritance pattern, i.e.paternal in *Actinidia* and maternal in *Clematoclethra*. The *clpP* gene was lacked in all the three sequenced cp genomes examined here indicating that the *clpP* gene loss is likely a conspicuous synapomorphic characteristic during the cp genome evolution of Actinidiaceae. Comprehensive sequence comparisons in Actinidiaceae cp genomes uncovered that there were apparently heterogenous divergence patterns among the cpDNA regions, suggesting a preferred data-partitioned analysis for cp phylogenomics. Twenty non-coding cpDNA loci with fast evolutionary rates are further identified as potential molecular markers for systematics studies of Actinidiaceae. Moreover, the cp phylogenomic analyses including 31 angiosperm plastomes strongly supported the monophyly of *Actinidia*, being sister to *Clematoclethra* in Actinidiaceae which locates in the basal asterids, Ericales.

## Introduction

Actinidiaceae as an economically important plant family includes the well-known kiwifruit species, i.e. *Actinidia chinensis* Planch. and *A*. *chinensis* var. *deliciosa* H.L.Li that can be eaten as fresh fruit or made into juice, jelly and dried fruit. There are *c*. 357 species included in three genera, i.e. *Actinidia* Lindl., *Clematoclethra* Maxim. and *Saurauia* Willd., in the kiwifruit family [1]. This family is widely distributed in Asia and the Americas displaying highly diverse morphological traits. In *Clematoclethra* species have bisexual flowers in one individual i.e. hermaphrodites, while in *Actinidia* species are dioecious or functionally dioecious [1–3]. The chloroplast (cp) genomes of *Clematoclethra* and *Actinidia* descend differently, that is with maternal and paternal transmission in *Clematoclethra* and *Actinidia* respectively [4]. However, currently little is known about the cp genomes evolution in these two closely related lineages.

The size of cp genomes in flowering plants ranges from 19 [5] to 218 kb [6], typically consisting of a pair of inverted repeats (IRs), a large single-copy (LSC) region and a small single-copy (SSC) region with the former one separating the latter two apart [7]. In spite of large degree conservation of organization and structure of the cp genomes in comparison to nuclear and mitochondrial genomes, gene losses and/or additions within cp genomes have been observed in several angiosperm lineages, e.g. orchids [8], Campanulaceae [9] and Rafflesiaceae [10]. Additionally, gene transfer between plastome, chondrome and nucleus has also been revealed in plants [11–13]. It has been proven that cp genome structural variations are accompanied by speciation over time, which likely provides evolutionary information [14]. Whether there are common structure variations in the cp genomes alongside the evolution of Actinidiaceae is so far little known.

Rates of nucleotide change between non-coding (intergenic regions and introns) and coding (exons of protein-coding genes) sequences can vary tremendously within a given taxonomic group [15–16]. As lack of phylogenetic information is one of the major problems leading to incompletely resolved phylogenetic trees [17], thus to generate maximum phylogenetic signal, the non-coding sequences should be especially considered, in particular when inferring phylogenies at lower taxonomic levels e.g. at generic level [18–19]. By far the phylogeny of Actinidiaceae, particularly the genus *Actinidia*, remains largely elusive and controversial [20–21]. Therefore, it is valuable to develop global non-coding molecular markers from Actinidiaceae plastomes to resolve phylogenetic relationships within this family (e.g. the genus *Actinidia*).

Genome skimming technique is so far one of the simplest and the most economical methodologies to obtain high-copy sequences [22–23], for instance plastome sequences, and to conduct intergeneric or family-wide phylogenomic analyses [24–26]. It is now convenient to obtain complete cp genome sequences and mine markers for plant systematics via genome skimming [27–28]. To date, there are few cp genomes that have been sequenced from basal asterids lineages [29–32]. As one of them, Actinidiaceae has only one species (*A*. *chinensis*) been sequenced the cp genomes [33]. Undoubtedly, more sequenced cp genomes from other species of Actinidiaceae will not only improve our understanding of the cp genome evolution in the kiwifruit family, but will also aid resolving the phylogenetic relationships of the Actinidiaceae, even of the asterids [34]. In this study, we generated and characterized the complete cp genomes of one representative of *Clematoclethra* (*C*. *lanosa*) and two species of *Actinidia* (*A*. *polygama* and *A*. *tetramera*) from Actinidiaceae using genome skimming approach. By applying comparative genomic analyses we aimed to 1) test whether the cp genomes of *Clematoclethra* and *Actinidia* evolved congruently in spite of their contrasting genetic patterns; 2) identify the most variable cp genome-wide markers for subsequent evolutionary studies of the kiwifruit family; and 3) uncover the potentiality of cp phylogenomic practice at varying taxonomic levels in asterids.

## Materials and Methods

### Plant materials and DNA sequencing

Fresh leaves of *Actinidia polygama* (ZXZ15021), *Actinidia tetramera* (ZXZ15014) and *Clematoclethra lanosa* (ZXZ15007) were collected from Huoditang forestry farm of Qinling Moutains in Shaanxi Province, China, in July 2015. Field studies were permitted and supported by Northwest A&F University to whom the Huoditang forestry farm belongs as an education and research experimental base. The voucher specimens of these three species were all deposited at the Trees Herbarium of Northwest A&F University.

Total genomic DNA was extracted from fresh leaf tissues of single individual per species using CTAB method [35]. Paired-end libraries (100 bp read length, insert size *c*. 500 bp) for each species were constructed from fragmented genomic DNA following standard Illumina protocols (Illumina, California, USA). DNAs from different species were indexed by barcodes and pooled together. Prepared libraries for all three species were finally sequenced in one lane on the Illumina HiSeq 2000 platform in Beijing Genomics Institute (BGI) in Shenzhen, China.

### Genome assembly, annotation and repeat analysis

Raw reads of each species were quality-filtered and *de novo* assembled using CLC Genomics Workbench v7.5 software (CLC Bio, Aarhus, Denmark). Contigs with length <300 bp and sequence coverage <50 were discarded [36]. The remaining contigs were analyzed by a BLAST search (http://blast.ncbi.nlm.nih.gov/) against the cp genome of *A*. *chinensis* [33] which was used as a reference in this study. Aligned contigs with ≥90% similarity and query coverage were designated as cp genomic contigs and ordered according to the reference. Small gaps were filled using trimmed Illumina reads, as conducted in Bock et al. [37] and Zhang et al. [26].

Initial cp genome annotation was performed using DOGMA software [38]. Start/stop codons and intron/exon boundaries were adjusted manually by comparing to the available *A*. *chinensis* cp genome [33]. tRNA genes were further conformed using tRNAscan-SE 1.21 [39]. OGDRAW program [40] was applied to graphically display the physical map of the circular cp genomes.

Tandem and palindromic repeats were determined by applying program REPuter [41]. Minimal tandem repeat size was set to 10 bp, intervening size between palindromic repeats constrained to a maximal length of 2 kb. Simple sequence repeats (SSRs) were detected using MISA perl script (http://pgrc.ipk-gatersleben.de/misa/). Minimum repeat unit was defined as 10 for mono-, 6 for di-, and 5 for tri-, tetra-, penta-, and hexanucleotide SSRs [42].

### Genome comparison and divergent hotspot identification

Chloroplast genome sequences of two additional Actinidiaceae species, i.e. *A*. *chinensis* (NC_026690) and *A*. *chinensis* var. *deliciosa* (NC_026691), were downloaded from GenBank. Together with our own sequenced cp genomes of *A*. *polygama*, *A*. *tetramera* and *C*. *lanosa*, we aligned these five cp genomes using MAFFT program [43] and manually adjusted where necessary. Full alignments with annotations were visualized using VISTA viewer [44] by referring to *A*. *chinensis* [33]. Multiple alignments of LSC, SSC, IRa (representing the two identical IRs), coding and non-coding regions in the matrix of five cp genomes were extracted respectively. Each region was compared to tally numbers of constant and variable alignment positions in MEGA v5.2 [45]. The number of nucleotide substitutions, in terms of single nucleotide polymorphisms (SNPs), divided by the total aligned length in each partition resulted in the percent variability for each region compared [19].

Five Actinidiaceae cp genomes were analysed to identify rapidly evolving molecular markers which can be used in subsequent phylogenetic studies. Both the coding and non-coding DNA fragments >200 bp in each cp genome were extracted separately by applying “Extract Sequences” option of DOGMA [38]. Then the homologous loci were aligned individually using MUSCLE [46] implemented in Geneious v9.0 [47] with default settings. Manual adjustments were made where necessary. The proportion of mutational events for each coding and non-coding loci was calculated as follows: the proportion of variation = (NS/L)*100, where NS = the number of nucleotide substitutions, L = the aligned sequence length.

### Phylogenomic analyses

Thirty-one complete cp genomes representing eight major lineages of the superasterids were included for phylogenomic analyses (S1 Table). *Arabidopsis thaliana* (L.) Heynh. and *Vitis vinifera* L. from superrosids were defined as outgroups according to previous studies [34, 48]. Given concerns about the likely effects of missing data on phylogenetic inferences [49], we analyzed the following two kinds of dataset accordingly: first, 79 plastid protein-coding unique genes of Actinidiaceae (S2 Table) were used, while that absent in other taxa were treated as missing data (“data-incomplete”); second, analyses based merely on the 56 common protein-coding genes (S3 Table) shared by all the representatives (S1 Table) were conducted (“data-complete”). Each gene was aligned individually using MUSCLE [46] in Geneious v9.0 [47], and then concatenated as a supermatrix for phylogenetic analyses. Gaps were not coded in both datasets.

Phylogenetic analyses of the concatenated datasets were carried out by maximum parsimony (MP), maximum likelihood (ML), and Bayesian inference (BI). MP analyses were implemented in PAUP* v4.0b10 [50]. Parsimony heuristic tree searches were performed with 1000 random addition sequence replicates, TBR branch swapping, MulTrees option in effect. Branch support (MPBS) was evaluated by 1000 bootstrap replicates [51].

ML and BI trees were inferred using RAxML v.8.2.8 [52] and MrBayes 3.2.6 [53] respectively in the CIPRES Science Gateway v3.3.3 [54], with concatenated supermatrix partitioned by genes. RAxML searches relied on the GTR + CAT model of nucleotide substitution, and 1,000 fast bootstrap ML reps were performed to assess the relative degree of support (MLBS) for internal nodes. In Bayesian analysis, two runs with four chains were run for 20,000,000 generations under the model GTR + G, with a sampling every 1,000 generations till convergence (the average standard deviation of split frequencies was less than 0.01). The first 25% of trees were discarded as burn-in, and the remaining trees were used to construct majority-rule consensus tree and estimate posterior probabilities (PP).

## Results

### Genome assembly and features

About 20 million reads were generated via genome skimming for each species. There were 516,840, 332,220, and 401,858 paired-end reads that were aligned for *A*. *polygama*, *A*. *tetramera* and *C*. *lanosa*, repsetively (Table 1). Based on *de novo* and reference-assisted assembly, complete cp genomes of the three species were obtained with high genome coverage (> 200×) (Table 1). The sequences of three cp genomes were deposited in GenBank (accession numbers: KX345297-KX345299).

**Table 1:** Statistic summary of the chloroplast genome sequencing, assembly and features.

	*Actinidia polygama*	*Actinidia tetramera*	*Clematoclethra lanosa*
Total paired-end reads	20,224,568	20,188,340	20,200,620
Aligned paired-end reads	516,840	332,220	401,858
Mean coverage	330.1	210.8	256.4
Size (bp)	156,583	157,659	156,731
LSC length (bp)	88,568	89,572	89,032
SSC length (bp)	20,397	20,497	20,399
IRs length (bp)	23,809	23,795	23,650
Number of genes	131	131	131
Protein-coding genes	83	83	83
Structure RNAs	48	48	48
GC content (%)	37.2	37.1	37.1
Coding regions (%)	49.4	49.3	50.0

**Fig 1:**
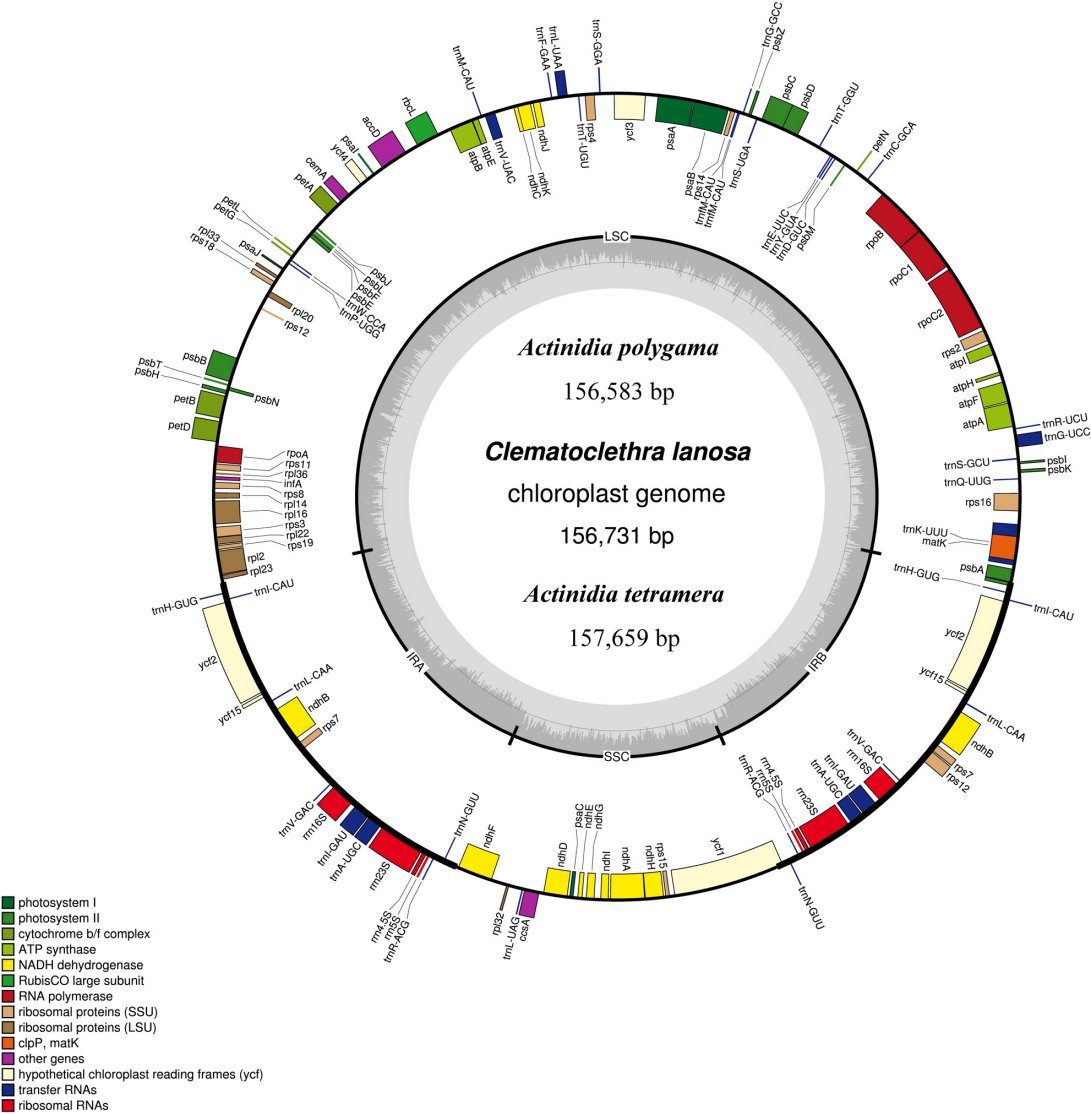
Gene map of *Clematoclethra lanosa* representing the chloroplast genome structure of three Actinidiaceae species. Genes drawn outside the circle are transcribed clockwise and those inside are counterclockwise. Genes belonging to different functional groups are color coded. The *darker gray* in the *inner circle* indicates the GC content of the chloroplast genome.

Eventually determined cp genome size of *A*. *polygama*, *A*. *tetramera* and *C*. *lanosa* was 156,583, 157,659 and 156,731 bp respectively (Table 1). All three complete cp genomes exhibited the typical quadripartite structure of land plants, consisting of a pair of IRs (23,650–23,809 bp) separated by one LSC (88,568–89,572 bp) and one SSC (20,397–20,497 bp) region (Table 1). An identical set of 131 genes with the same linear order were encoded in three cp genomes. Here we reported the cp genome map of *C*. *lanosa* as the representative (Fig 1). 113 genes are unique, of which 79 are protein-coding genes (Fig 1). The *clpP* gene was undetectable in both *Clematoclethra* and *Actinidia* cp genomes (Fig 1, S2 Table). Among the 11 intron-containing genes (*atpF*, *ndhA*, *ndhB*, *petB*, *petD*, *rpl2*, *rpl16*, *rpoC1*, *rps12*, *rps16*, *ycf3*), only *rps12* and *ycf3* contained two intervening introns, while others contained one intron each. The boundaries between IRs and single copy regions were conserved in *Clematoclethra* and *Actinidia* cp genomes, with *ycf1* and *psbA* extending into the IRb (Fig 1).

**Fig 2:**
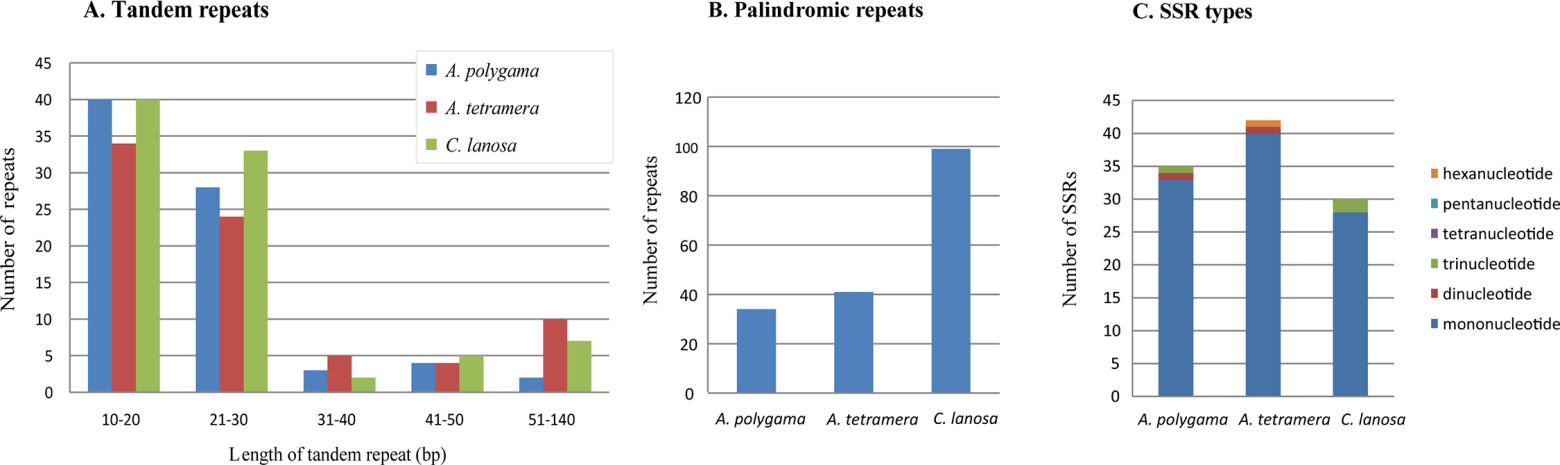
Repeat analyses. (A) Histogram showing the number of tandem repeats in the three Actinidiaceae chloroplast genomes. (B) Summary of palindromic repeats in each chloroplast genome. (C) SSR unit size distribution in the three chloroplast genomes.

Three classes of repeats (tandem, palindromic and SSRs) were investigated in our sequenced cp genomes. Tandem repeats that ranged from 10 to 20 bp were the most abundant, that had their sizes between 20 and 30 bp were secondarily abundant, while that with lengths greater than 30 bp were much less in all three species (Fig 2A). Palindromic repeats were much more in the cp genome of *C*. *lanosa* (99) than that in two *Actinidia* species (34 and 41 respectively) (Fig 2B). Analysis of occurrence and type of SSRs indicated that four categories of SSRs, i.e. mono-, di-, tri-, and hexa-nucleotide were detected. Mononucleotide repeats were the most dominant, while other types were far less in the cp genomes of three species (Fig 2C). Not all the SSRs mentioned above occurred in all the species, that is, there were three types in *A*. *polygama* (mono-, di- and tri-nucleotide) and *A*. *tetramera* (mono-,di- and hexa-nucleotide), but only two types in *C*. *lanosa* (mono- and tri-nucleotide) (Fig 2C).

### Sequence divergence estimation and molecular marker identification

**Fig 3:**
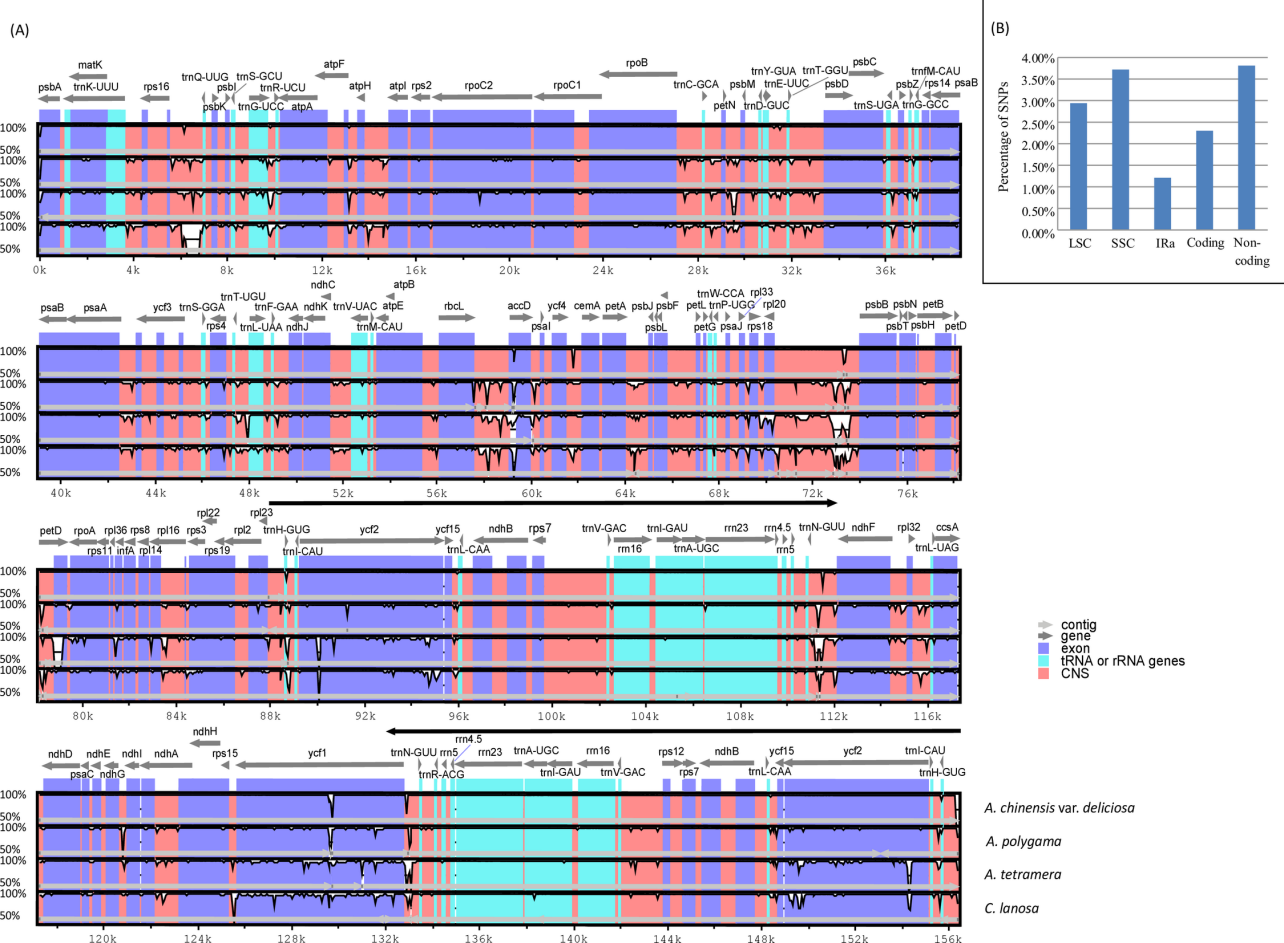
Heterogenous sequence divergence of the five Actinidiaceae chloroplast genomes. (A) VISTA-based identity plots of Actinidiaceae chloroplast genomes with *A*. *chinensis* used as a reference. Thick arrowed black lines show the inverted repeat regions (IRs) in the chloroplast genome. All the chloroplast genome regions are color coded as protein coding, rRNA, tRNA or conserved non-coding sequences (CNS). (B) Varied sequence divergence among the LSC, SSC and IRs (in terms of IRa), as well as the coding and non-coding regions. The percentage of nucleotide substitutions (SNPs) for each region was estimated respectively.

Sequence divergences among the five Actinidiaceae species (*A*. *chinensis*, *A*. *chinensis* var. *deliciosa*, *A*. *polygama*, *A*. *tetramera* and *C*. *lanosa*) were visually quantified by “percent identity plots” and estimated by the percentage of nucleotide substitutions (SNPs) (Fig 3). Global alignments further confirmed that the five cp genomes of Actinidiaceae were perfectly syntenic (Fig 3A). Across the whole cp genomes, we found that sequence variations were not uniform but highly heterogenous among different regions (Fig 3A). The estimation of the percentage of SNPs in LSC, SSC and IRs (here in terms of IRa) regions suggested that sequences of the IRs diverged at slower rates (1.21%) than that of their adjacent single-copy regions, LSC (2.94%) and SSC (3.92%) (Fig 3B). Coding regions (2.30%) were more conserved than non-coding regions (3.81%) as expected (Fig 3B).

**Fig 4:**
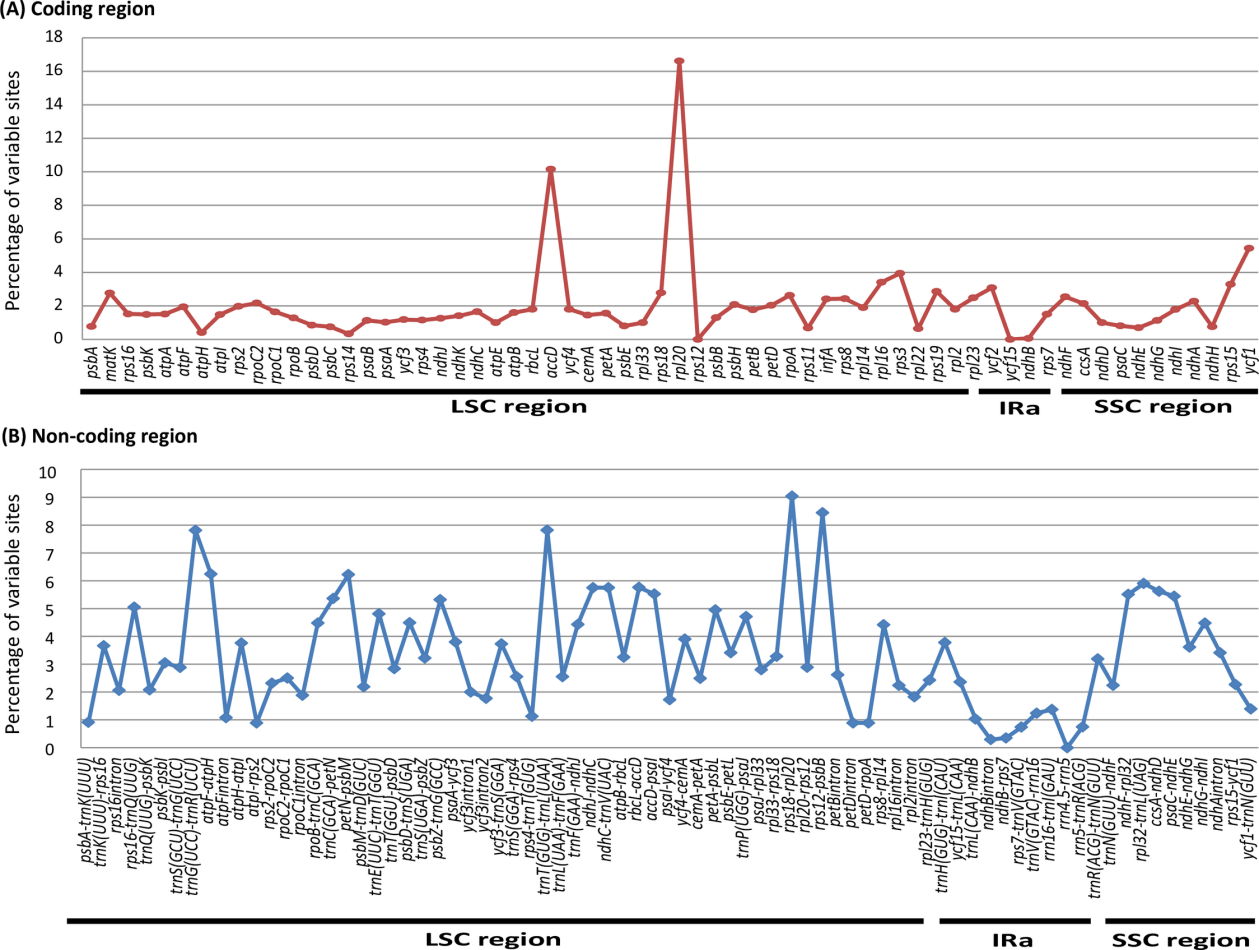
Percentage of variable characters (SNPs) in homologous loci among five chloroplast genomes of Actinidiaceae. (A) Coding region. (B) Non-coding region. The homologous loci are oriented according to their locations in the chloroplast genome.

Distribution patterns of variable characters (uninformative + informative SNPs) differed largely in coding and non-coding loci (>200 bp) in these five Actinidiaceae cp genomes (Fig 4). It showed that the proportion of variability in non-coding loci ranged from 0.00% to 9.04% with mean value as 3.36% which was 1.6 times greater than that in the coding loci (2.01% on average) (Fig 4). Variations in most (57 out of 65) of the coding DNA fragments were less than 3% (Fig 4A). Only three genes i.e. *rpl20*, *accD* and *ycf1* had their variations exceeded 5%. In contrast, there were 32 non-coding loci showed relative high degree of divergence (above 3.6%, Fig 4B). Considering the relatively high percentage of variations and the convenience of universal primers developing for PCR and sequencing experiments, we chose 20 highly variable intergenic loci as potential molecular markers for subsequent phylogenetic studies (Table 2). Percentage of variations of these 20 non-coding loci all exceeded 3.7%, among which 16 had a percentage of variable characters (VCs) greater than 4% (Table 2).

**Table 2:** Twenty plastid non-coding loci with high genetic divergence identified in Actinidiaceae.

Region	Length (bp)	Aligned length (bp)	No. VCs	Percentage of VCs (%)	No. PICs	Percentage of PICs (%)
*rps18-rpl20*	283–326	332	30	9.04	9	2.71
*trnT*(GUG)*-trnL*(UAA)	523–636	639	50	7.82	27	4.23
*trnG*(UCC)*-trnR*(UCU)	341–408	448	35	7.81	8	1.79
*atpF-atpH*	397–437	449	28	6.24	11	2.45
*petN-psbM*	609–754	756	47	6.22	14	1.85
*rpl32-trnL*(UAG)	760–787	813	48	5.90	6	0.74
*ndhC-trnV*(UAC)	921–1200	1234	71	5.75	15	1.22
*ndhF-rpl32*	735–953	980	54	5.51	8	0.82
*trnC*(GCA)-*petN*	712–743	765	41	5.42	8	1.06
*rps16-trnQ*(UUG)	869–1478	1544	78	5.05	11	0.72
*petA-psbL*	963–978	1010	50	4.95	11	0.73
*trnE*(UUC)*-trnT*(GGU)	793–823	832	40	4.81	7	0.84
*trnP*(UGG)*-psaJ*	398–400	403	19	4.71	5	1.24
*rpoB-trnC*(GCA)	1139–1265	1271	57	4.48	7	0.55
*ndhG-ndhI*	264–357	357	16	4.48	5	1.40
*trnF*(GAA)*-ndhJ*	692–714	722	32	4.43	11	1.52
*ycf4-cemA*	653–950	951	37	3.89	6	0.63
*psaA-ycf3*	730–749	764	29	3.80	15	1.96
*atpH-atpI*	1107–1149	1196	45	3.76	8	0.67
*ycf3-trnS*(GGA)	823–831	832	31	3.73	8	0.96

VCs: variable characters; PICs: parsimony informative characters.

### Phylogenomic reconstruction

**Fig 5:**
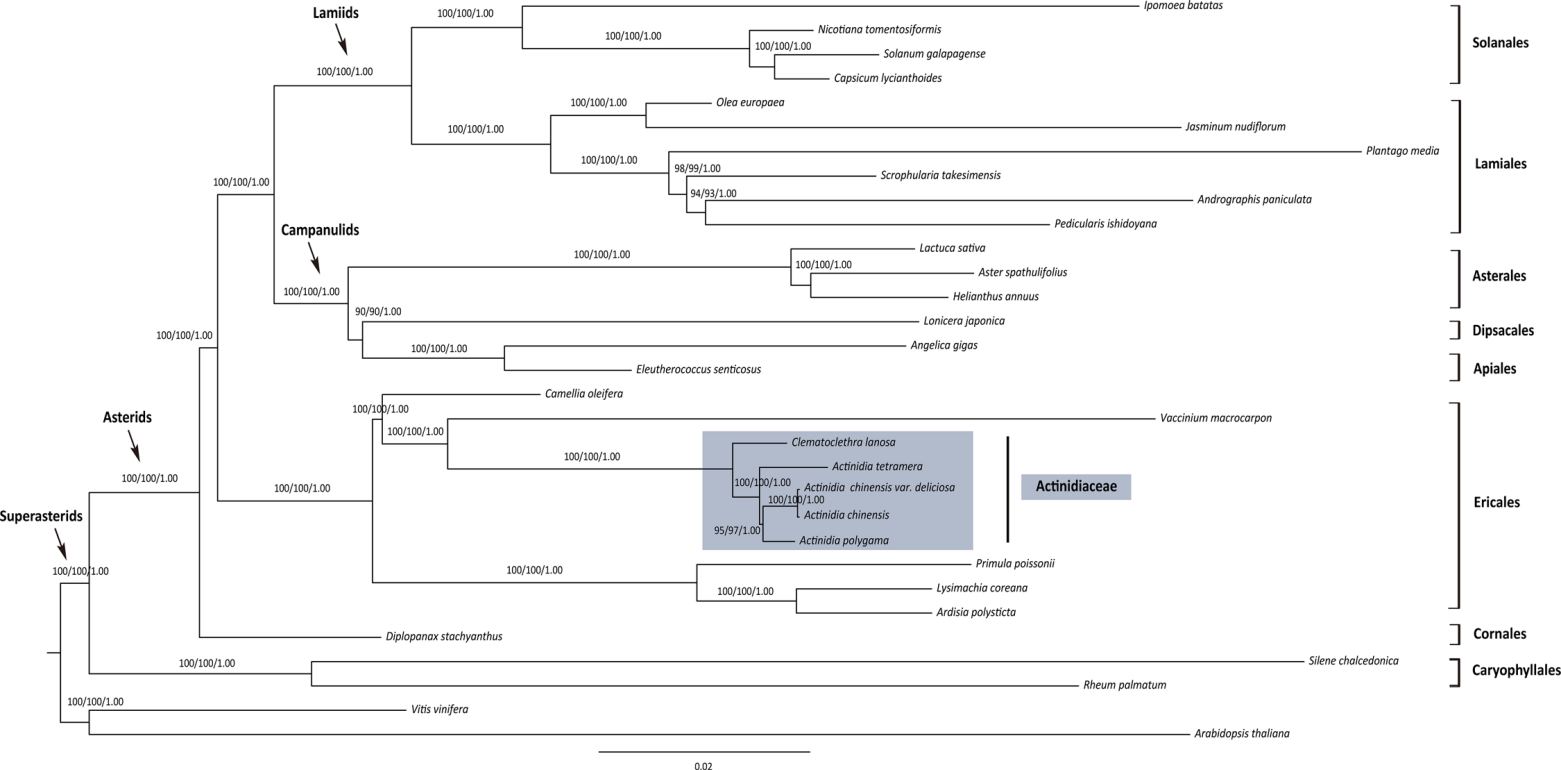
Maximum likelihood (ML) tree for 31 taxa based on 56 common plastid protein-coding genes (“data-complete”). Values above the branches represent maximum parsimony bootstrap (MPBS)/maximum likelihood bootstrap (MLBS)/Bayesian inference posterior probability (PP). The Actinidiaceae lineage is indicated in shaded.

The final length of the aligned matrix for the “data-incomplete” and “data-complete” data sets contained 91,222 and 58,876 unambiguously aligned nucleotide characters respectively. Because these two data sets generated congruent phylogenetic trees, we here only reported the results based on the 56 common protein-coding plastid genes (“data-complete” data set) as shown in Fig 5. Asterids was highly supported as monophyletic (100/100/1.00) and the sister relationship of lamiids and campanulids was well supported (100/100/1.00). Cornales was resolved as the basal most group in the asterids (100/100/1.00), and the Ericales diverged subsequently (100/100/1.00). Caryophyllales was revealed as the sister to asterids in the superasterids clade (100/100/1.00). The sister relationship of Ericales and core asterids (lamiids + campanulids) also obtained high support values (100/100/1.00).

The Actinidiaceae were recovered as a monophyly in Ericales with high statistic supports (100/100/1.00). The phylogenomic tree indicated a branching order of Actinidiaceae, Ericaceae, Primulaceae and Theaceae as (((Actinidiaceae, Ericaceae), Theaceae), Primulaceae) in Ericales, each node getting high support values (100/100/1.00). The sampled four *Actinidia* taxa clustered in a clade with high statistic supports (100/100/1.00), being sister to *Clematoclethra*. *A*. *tetramera* diverged firstly (95/97/1.00), followed by *A*. *polygama* (100/100/1.00) in the *Actinidia* lineage. As expected, *A*. *chinensis* and *A*. *chinensis* var. *deliciosa* were closely related (100/100/1.00).

## Discussion

### The conserved chloroplast genomes of *Clematoclethra* and *Actinidia*

Selective pressure leads to divergent traits during plant evolution, which may be tracked in chloroplast genomes, for instance, the non-photosynthetic lifestyle of certain plant groups such as *Epipogium aphyllum* and *Epipogium roseum* [5], *Orobanche* [13], and *Petrosavia stellaris* [55] tends to be associated with the enormously reduced cp genome size. Complete sequencing and comparative analyses here revealed that the *Clematoclethra* species (*C*. *lanosa*) bears a highly conserved cp genome in terms of its architecture and linear sequence order with either the two newly sequenced *Actinidia* (*A*. *polygama* and *A*. *tetramera*) in this study or the published *A*. *chinese* cp genome [33]. Chloroplast genome size of *C*. *lanosa* (156.7 kb) is similar to that of *A*. *polygama* (156.6 kb) and *A*. *tetramera* (157.7 kb) (Table 1), showing a typical cp genome size range (115 to 165 kb) of angiosperms [56]. Although the different inheriting patterns of the *Actinidia* and *Clematoclethra* cp genomes, i.e. paternal passing in *Actinidia* and maternal in *Clematoclethra* [4], no significant structural variation was observed (Figs 1 and 3A). The gene content, gene order, GC content, size of IRs regions, and junctions of single copy and IR regions (Table 1, Fig 1) all indicate highly syntenic nature of the cp genomes in the two closely related genera within Actinidiaceae.

*clpP* gene was completely lost in the sequenced cp genomes of *Actinidia* [this study, 33] and also was absent in *Clematoclethra* (*C*. *lanosa*). The lack of certain plastid genes is not unusual since previous studies illustrated that several cp genes have been lost in certain groups, such like, the *clpP* gene in *Primula poissonii* [31] and *Camellia oleifera* [32], the *accD* gene in *Trifolium* [57], the *rpl32* gene in *Populus* [58], and the *infA* gene in rosids [59]. The *clpP* gene loss detected in both *Clematoclethra* and *Actinidia* is most likely a conspicuous common feature during their cp genomes evolution, suggesting that the *clpP* gene loss event might happen in their common ancestor. More species from the kiwifruit family, especially representatives of the third genus *Saurauia*, will be sequenced to determine whether *clpP* gene loss is synapomorphy of the Actinidiaceae.

Occurrences and types of tandem repeats are quite similar with approximately the same levels in each cp genomes (Fig 2A). However, the palindromic repeats in the cp genome of *C*. *lanosa* were more than that in two *Actinidia* species (Fig 2B). Different abundance of palindromic repeats in the cp genomes of *Clematoclethra* and *Actinidia* would likely provide additional evolutionary information since presence and abundance of repetitive sequences in cp or nuclear genomes may contain phylogenetic signal [16, 60]. Mononucleotide SSRs are abundant in all the three cp genomes (Fig 2C), which can be used as potential cpSSR markers in studies on population genetics of kiwifruit species [33].

### Heterogenous divergence patterns in chloroplast genomes and molecular markers development

Alignment of our three cp genomes and two other published cp genomes of Actinidiaceae (*A*. *chinensis* and *A*. *chinensis* var. *deliciosa*) [33] uncovered obvious heterogenous sequence divergence within the Actinidiaceae cp genomes (Fig 3A). The single-copy regions (LSC and SSC) show a higher sequence divergence than that in the inverted repeat regions (in terms of IRa) (Fig 3B). This is possible because the gene conversion of IRs constrained the nucleotide variations of inverted repeat sequences [61], which results in the lower genetic divergence of IRs. The coding regions are more conserved than non-coding regions (Fig 3B), likely resulting from the strong selective pressure on the function constraint of coding sequences [62]. Different regions of cp genomes display varied evolutionary rates reflecting that data-partitioned cp phylogenomic analysis such as partitioning cp genomes data by LSC, SSC and IRs [16], by coding and non-doing regions [63] or by genes [27–28], may be more reasonable in the Actinidiaceae cp phylogenomic practice.

Sequence variation analyses of the coding and non-coding loci (longer than 200 bp) across the five examined cp genomes indicate that each locus shows independent/distinct evolutionary patterns (Fig 4) as revealed in Poaceae [64], mimosoid legume [65] and *Silene* [66]. In general, coding genes would have their sequence variations (percentage of variable characters) lower than 4% but some exceptions are observed here, for instance, *accD* (in the LSC region), *rpl20* (in the LSC region) and *ycf1* (in the SSC region). These exceptions display more SNPs than all other coding loci particularly with *rpl20* as one which varies the most quickly. There have been some observations of accelerated variation rate of the plastid genes, such as *psb* in Poaceae [67], *rps* in Saxifragales [68], and *rps* and *clpP* genes in Caryophyllales [69]. Nevertheless, those speeded divergence observations were thought as results of abnormal DNA replication, repair or recombination [65, 70]. It is likely that the heterogenous divergences among plastid genes in Actinidiaceae probably are driven by similar mechanism as in the above described groups.

Chloroplast DNA fragments such like *matK*, *psbC*-*trnS*, *rbcL*, and *trnL*-*trnF* have been used most commonly in *Actinidia* phylogenetic studies [20–21, 71], but the resulted trees have not been resolved clearly. The difficulty mainly lies in the lack of phylogenetic information due to the low sequence variation of commonly used genetic markers [21]. Therefore, we need to develop rapidly evolving genetic markers for the phylogenetic study of Actinidiaceae, particularly of the genus *Actinidia*, which will shed light on our understanding of the evolutionary history of this valuable economical plant group. The average proportion of variability in non-coding loci is higher (3.36%) than that in the coding loci (2.01%) (Fig 4). In addition, it is convenient to design universal PCR and Sanger sequencing primers for non-coding DNA fragments with length from *c*. 300 bp to 1,500 bp by using the adjacent conserved coding sequences [18–19]. We thus choose 20 highly variable non-coding cpDNA loci as potential molecular markers (Table 2) for subsequent phylogenetic studies of the kiwifruit family. Some of these loci such as *trnT*(UGU)-*trnL*(UAA) and *rpl32*-*trnL*(UAG) has been used in phylogenetic studies of intractable plant groups, e.g. in woody bamboos (Poaceae, Bambusoideae) with relatively robust solutions [72]. These 20 cp genome-wide intergenic loci will probably provide sufficient phylogenetic information for the reconstruction of the phylogeny of Actinidiaceae.

### Chloroplast phylogenomic practice

The presence of missing data is not unusual in phylogenomic analyses, though its impact on phylogenetic evaluation has been widely debated [73–76]. Using our sequenced three cp genomes via genome skimming together with 28 other publically available plastomes data (S1 Table), we reconstructed the phylogeny of Ericales and its related groups. The “data-incomplete” and “data-complete” data sets give rise to a congruent topology of the 31 sampled taxa with high level of confidence (Fig 5, from the “data-complete” data set), which indicates that the relatively small missing data (23 out of 79 plastid genes) probably have no ill impact on the phylogenetic inferences.

The relationships derived from our phylogenomic analyses among the major lineages of asterids, i.e. Cornales, Ericlaes and core asterids (lamiids + campanulids), are highly supported as stated previously [34, 46, 77]. The branching orders of species within lamiids and campanulids are consistent with recent studies [78–79] as ((Solanales, Lamiales), ((Dipsacales, Apiales), Asterales)). Our analyses also recover the phylogenetic relationships of four sampled families in Ericales as (((Actinidiaceae, Ericaceae), Theaceae), Primulaceae), in agreement with the result of Yao et al. [33]. The Actinidiaceae is a monophyly with the sampled four *Actinidia* taxa clustering in a clade as sister to *Clematoclethra* (Fig 5). The sister relationship of *A*. *chinensis* and *A*. *chinensis* var. *deliciosa* is consistent with the results of previous studies [20, 71]. Moreover, phylogenetic placements of *A*. *polygama* and *A*. *tetramera* are also resolved with high supports within *Actinidia*.

To date, the genome skimming method has been thought as an economical and advanced technique with great potential not only in the plastomes but also for the mitochondrial and nuclear genomes studies [80–83]. Chloroplast genome has been broadly employed as a routine practice in the plant systematics because of its essentially recombination-free and high degree of identical copies per cell [84]. Applying genome skimming on the expanded taxon samples will facilitate the phylogenomic study of the large asterids clade in angiosperm.

## Conclusions

In summary, in the present study we generated cp genomes of three species from the family Actinidiaceae, including one species of *Clematoclethra* and two species of *Actinidia*, using genome skimming approach. These finished cp genomes will facilitate the development of plastid genetic engineering for these valuable fruit species and provide additional information about the evolutionary history of cp genomes in the whole kiwifruit family. The cp genomes of *Clematoclethra* and *Actinidia* are highly conserved in the structure and content in spite of their contrasting genetic processes of plastomes. The *clpP* gene loss likely provides a prominent synapomorphic characteristic of Actinidiaceae. Obviously heterogenous divergence patterns in different regions of the Actinidiaceae cp genomes are revealed, according to which 20 rapidly evolving intergenic loci are identified as potential cp genome-wide markers for subsequent phylogenetic studies of this family. The Actinidiaceae and its subordinate genera are both strongly supported as monophyletic located in the Ericales in basal asterids. The phylogenetic relationships within the large asterids clade, even within the enigmatical genus *Actinidia*, have been robustly resolved based on the cp genomes data, suggesting the bright probability of cp phylogenomics for tackling difficult phylogenies in flowering plants.

## Supporting Information

S1 Table Taxa and GenBank accession numbers included in the phylogenomic analyses. (DOCX)

S2 Table List of 79 unique plastid genes of Actinidiaceae included in the “data-incomplete” data set of phylogenomic analyses. (DOCX)

S3 Table List of 56 common unique plastid genes included in the “data-complete” data set of phylogenomic analyses. (DOCX)
