# Supplementary material for: Chloroplast Genome Evolution in Actinidiaceae: clpP Loss, Heterogenous Divergence and Phylogenomic Practice
Source: PLoS One. 2016 Sep 2;11(9):e0162324. doi: 10.1371/journal.pone.0162324 (PMC5010200; doi:10.1371/journal.pone.0162324)
Supplement: S1 Table — (DOCX) [file pone.0162324.s001.docx]

**Table S1. Taxa and GenBank accession numbers included in the phylogenomic analyses.**

| Classification | Taxon | GenBank |
| --- | --- | --- |
| Ericales/Actinidiaceae  Ericales/Ericaceae  Ericales/Primulaceae  Ericales/Theaceae  Lamiales/Acanthaceae  Lamiales/Plantaginaceae  Lamiales/Oleaceae  Lamiales/Orobanchaceae  Lamiales/Scrophulariaceae  Solanales/Convolvulaceae  Solanales/Solanaceae  Asterales/Compositae  Apiales/Apiaceae  Dipsacales/Caprifoliaceae  Cornales/Cornaceae  Caryophyllales/Polygonaceae  Caryophyllales/Caryophyllaceae  Brassicales/Brassicaceae  Vitales/Vitaceae | *Actinidia chinensis*  *A. chinensis* var. *deliciosa*  *A. polygama*  *A. tetramera*  *Clematoclethra lanosa*  *Vaccinium macrocarpon*  *Ardisia polysticta*  *Lysimachia coreana*  *Primula poissonii*  *Camellia oleifera*  *Andrographis paniculata*  *Plantago media*  *Jasminum nudiflorum*  *Olea europaea*  *Pedicularis ishidoyana*  *Scrophularia takesimensis*  *Ipomoea batatas*  *Capsicum lycianthoides*  *Nicotiana tomentosiformis*  *Solanum galapagense*  *Aster spathulifolius*  *Helianthus annuus*  *Lactuca sativa*  *Angelica gigas*  *Eleutherococcus senticosus*  *Lonicera japonica*  *Diplopanax stachyanthus*  *Rheum palmatum*  *Silene chalcedonica*  *Arabidopsis thaliana*  *Vitis vinifera* | NC_026690.1  NC_026691.1  KX345297^*^  KX345298^*^  KX345299^*^  NC_019616.1  NC_021121.1  NC_026197.1  NC_024543.1  NC_023084.1  NC_022451.2  NC_028520.1  NC_008407.1  NC_013707.2  NC_029700.1  NC_026202.1  NC_026703.1  NC_026551.1  NC_007602.1  NC_026878.1  NC_027434.1  NC_007977.1  NC_007578.1  NC_029393.1  NC_016430.1  NC_026839.1  NC_029750.1  NC_027728.1  NC_023359.1  NC_000932.1  NC_007957.1 |

* The newly sequenced chloroplast genomes in the current study.
