# Supplementary material for: Chloroplast Genome Evolution in Actinidiaceae: clpP Loss, Heterogenous Divergence and Phylogenomic Practice
Source: PLoS One. 2016 Sep 2;11(9):e0162324. doi: 10.1371/journal.pone.0162324 (PMC5010200; doi:10.1371/journal.pone.0162324)
Supplement: S2 Table — (DOCX) [file pone.0162324.s002.docx]

**Table S2. List of 79 unique plastid genes of Actinidiaceae included in the “data-incomplete” data set of phylogenomic analyses.**

*accD, atpA, atpB, atpE, atpF, atpH, atpI, ccsA, cemA, infA, matK, ndhA, ndhB, ndhC, ndhD, ndhE, ndhF, ndhG, ndhH, ndhI, ndhJ, ndhK, petA, petB, petD, petG, petL, petN, psaA, psaB, psaC, psaI, psaJ, psbA, psbB, psbC, psbD, psbE, psbF, psbH, psbI, psbJ, psbK, psbL, psbM, psbN, psbT,
psbZ, rbcL, rpl14, rpl16, rpl2, rpl20, rpl22, rpl23, rpl32, rpl33, rpl36, rpoA, rpoB, rpoC1, rpoC2, rps2, rps3, rps4, rps7, rps8, rps11, rps12, rps14, rps15, rps16, rps18, rps19, ycf1, ycf15, ycf2, ycf3,
ycf4*
