# Supplementary material for: Chloroplast Genome Evolution in Actinidiaceae: clpP Loss, Heterogenous Divergence and Phylogenomic Practice
Source: PLoS One. 2016 Sep 2;11(9):e0162324. doi: 10.1371/journal.pone.0162324 (PMC5010200; doi:10.1371/journal.pone.0162324)
Supplement: S3 Table — (DOCX) [file pone.0162324.s003.docx]

**Table S3. List of 56 common unique plastid genes included in the “data-complete” data set of phylogenomic analyses.**

*atpA, atpE, atpF, atpH, atpI, cemA, matK, ndhE, petA, petB, petD, petG, petL, petN, psaA, psaB, psaC, psaI, psaJ, psbA, psbC, psbD, psbE, psbF, psbH, psbI, psbJ, psbK, psbL, psbM, psbN, psbT, rbcL, rpl14, rpl16, rpl2, rpl22, rpl32, rpl33, rpl36, rpoB, rpoC1, rpoC2, rps2, rps3, rps4, rps7, rps8, rps11, rps14, rps15, rps16, rps18, ycf2, ycf3, ycf4*
